# Retraction: *In vitro* anticancer effects of JI017 on two prostate cancer cell lines involve endoplasmic reticulum stress mediated by elevated levels of reactive oxygen species

**DOI:** 10.3389/fphar.2026.1880306

**Published:** 2026-05-27

**Authors:** Frontiers Editorial Office

The journal retracts the 13 May 2021 article cited above.

Following publication, concerns were raised regarding the integrity of the images in the published figures. Image duplication concerns were identified in Figure 2B.

The authors and their institution have remained unresponsive to multiple requests for the raw data for this study. Given extant concerns over the validity of the study, the article has been retracted.

This retraction was approved by the Chief Editors of Frontiers in Pharmacology and the Chief Executive Editor of Frontiers. The authors have not responded to correspondence regarding this retraction.

Frontiers would like to thank Sholto David for contacting the journal regarding the published article.

